# Ultrastructural Characteristics of DHA-Induced Pyroptosis

**DOI:** 10.1007/s12017-019-08586-y

**Published:** 2020-01-04

**Authors:** Deron R. Herr, Ting Yu Amelia Yam, Wan Shun Daniel Tan, Sally Shuxian Koh, Wai Shiu Fred Wong, Wei-Yi Ong, Kanokporn Chayaburakul

**Affiliations:** 1 Department of Pharmacology, Yong Loo Lin School of Medicine, National University of Singapore, Singapore, 119260 Singapore; 2 Department of Anatomy, Yong Loo Lin School of Medicine, National University of Singapore, Singapore, 119260 Singapore; 3 Immunology Program, Life Science Institute, National University of Singapore, 28 Medical Drive, Singapore, 117456 Singapore; 4 Singapore-HUJ Alliance for Research and Enterprise, National University of Singapore, 1 CREATE Way, Singapore, 138602 Singapore; 5 Anatomy Unit, Faculty of Science, Rangsit University, Pathumthani, 12000 Thailand

**Keywords:** Docosahexaenoic acid (DHA), Pyroptosis, Scanning electron microscopy (SEM), Transmission electron microscopy (TEM), Membrane pores, Microglia

## Abstract

Microglial cells are resident macrophages of the central nervous system (CNS) that respond to bioactive lipids such as docosahexaenoic acid (DHA). Low micromolar concentrations of DHA typically promote anti-inflammatory functions of microglia, but higher concentrations result in a form of pro-inflammatory programmed cell death known as pyroptosis. This study used scanning electron microscopy (SEM) and transmission electron microscopy (TEM) to investigate the morphological characteristics of pyroptosis in BV-2 microglial cells following exposure to 200 µM DHA. Vehicle-treated cells are characterized by extended processes, spine-like projections or 0.4 to 5.2 µm in length, and numerous extracellular vesicles (EVs) tethered to the surface of the plasma membrane. In contrast to vehicle-treated cells, gross abnormalities are observed after treating cells with 200 µM DHA for 4 h. These include the appearance of numerous pits or pores of varying sizes across the cell surface, structural collapse and flattening of the cell shape. Moreover, EVs and spines were lost following DHA treatment, possibly due to release from the cell surface. The membrane pores appear after DHA treatment initially measured ~ 30 nm, consistent with the previously reported gasdermin D (GSDMD) pore complexes. Complete collapse of cytoplasmic organization and loss of nuclear envelope integrity were also observed in DHA-treated cells. These processes are morphologically distinct from the changes that occur during cisplatin-induced apoptosis, such as the appearance of apoptotic bodies and tightly packed organelles, and the maintenance of EVs and nuclear envelope integrity. Cumulatively, this study provides a systematic description of the ultrastructural characteristics of DHA-induced pyroptosis, including distinguishing features that differentiate this process from apoptosis.

**Electronic supplementary material:**

The online version of this article (10.1007/s12017-019-08586-y) contains supplementary material, which is available to authorized users.

## Background

Pyroptosis is an inflammatory form of programmed cell death (PCD) typically associated with an antimicrobial response to infection by intracellular pathogens. This process is triggered by recognition of conserved microbial features by host pattern recognition receptors (PRRs) that are expressed by immune antigen-presenting cells of the monocyte/macrophage lineage (Liu and Lieberman 2017). This leads to activation of protein complexes known as inflammasomes (Poh et al. 2019), and activation of inflammatory caspases, including caspase-1 and caspase-11 (caspase-4/-5 in humans) (Miao et al. 2011; Shi et al. 2014). The result is cleavage of a cellular substrate called gasdermin D (GSDMD) to produce an N-terminal fragment capable of forming pores in the plasma membrane (Aglietti et al. 2016; Ding et al. 2016; Liu et al. 2016; Sborgi et al. 2016). During pyroptosis, immune cells recognize foreign danger signals, release pro-inflammatory cytokines, swell, burst and die. The released cytokines attract other immune cells, further propagating the inflammatory response. Pyroptosis promotes the rapid clearance of various bacterial and viral infections by removing intracellular replication niches and enhancing the host’s defensive responses. Besides being an important component of the innate immune system, pyroptosis has also been shown to be involved in sterile inflammatory processes (Barrington et al. 2017; Poh et al. 2019).

In contrast to pyroptosis, apoptosis is an immunologically ‘silent’, non-inflammatory form of PCD that can occur either via an extrinsic or an intrinsic pathway. The extrinsic pathway is activated by signaling through cell surface death receptors, whilst the intrinsic pathway is activated by mitochondrial damage. Both pathways lead to the activation of executioner caspases (caspase-3, 6, and 7), which target a large number of substrates to produce apoptosis (Taylor et al. 2008). Pyroptosis is also distinguished from a third form of PCD, necroptosis. The latter is triggered by activation of receptor-interacting protein kinase-3 (RIPK3), which phosphorylates the pseudokinase MLKL, causing it to translocate to the plasma membrane to induce cell permeabilization (Linkermann and Green 2014). The above forms of PCD are distinct from acute cell breakdown due to the direct action of a damaging stimulus, since the latter requires no cellular activity and is prevented only by the absence of the damaging stimulus (Jaattela 2002; Fink and Cookson 2005).

Cells undergoing apoptosis or pyroptosis have distinct morphological features. In the initial phases, apoptotic cells shrink, whereas pyroptotic cells are characterized by pronounced swelling (Fink and Cookson 2006). In addition, the nuclear morphology of apoptotic cells is characterized by pyknosis (the irreversible condensation of marginated chromatin), as well as DNA fragmentation (Majno and Joris 1995). By contrast, the nuclei of pyroptotic cells show chromatin condensation, but without DNA fragmentation (Zychlinsky et al. 1992; Watson et al. 2000). Lastly, apoptosis and pyroptosis have differential effects on the plasma membrane. While apoptosis causes the formation of membrane blebs of 1–5 µm in diameter, or apoptotic bodies (Zhang et al. 2018), pyroptosis results in the formation of 15–32 nm pores and general swelling of the membrane (Liu et al. 2016).

Microglia are resident cells of the CNS that regulate brain development, maintenance of neuronal networks, and injury repair. They serve as brain macrophages for the elimination of microbes, dead cells, redundant synapses, protein aggregates, and other particulate and soluble antigens that may endanger the CNS (Colonna and Butovsky 2017). Our recent study revealed that 30 µM DHA could stimulate anti-inflammatory effects, but > 50 µM DHA-induced pyroptosis in BV-2 microglial cell line, as evidenced by cell swelling morphology and decreased viability. DHA-treated cells also exhibited the molecular hallmarks of pyroptosis, including increased expression of inflammatory cytokines and activation of caspase-1 activity (Srikanth et al. 2018).

Recent studies have begun to address the role of microglial pyroptosis in CNS injury, e.g., in pathophysiology of traumatic brain injury (Lee et al. 2019). Thus far, however, no systematic ultrastructural characterization has yet been carried out to confirm the presence of the canonical features of pyroptosis in microglia. In the current study, we used phase-contrast microscopy, scanning electron microscopy, and transmission electron microscopy to confirm and characterize the pyroptotic phenotype induced in BV-2 microglial cells after exposure to DHA.

## Materials and Methods

### Chemicals and Cell Culture

DHA (cat #D2534) and cis-Diammineplatinum(II) dichloride (cisplatin, cat #P4394) were purchased from Sigma-Aldrich (USA). DHA was prepared by solubilizing in methanol, drying aliquots under vacuum, and storing the pellets under inert gas at − 30 °C. Pellets were resuspended in sterile-filtered 10% fatty acid-free bovine serum albumin at 20 mM and further diluted with Dulbecco’s modified Eagle’s medium (DMEM) immediately before use. Cisplatin was prepared fresh weekly by dissolving in dimethyl sulfoxide at 40 mM. Further dilutions were made in DMEM when needed for experiments. BV-2 immortalized mouse microglial cells (Blasi et al. 1990) were maintained as a monolayer culture on tissue culture dishes at 37 °C, 5% CO_2_, 100% humidity in DMEM supplemented with 5% heat-inactivated fetal bovine serum and antibiotics. All experiments were performed on cells within six passages of recovery from cryopreservation. Cells were grown on culture dishes or on poly-l-lysine-coated glass coverslips for 48 h prior to experiments. Treatments were performed by introducing DHA or cisplatin to the indicated concentrations without prior media change or serum deprivation.

### Scanning Electron Microscopy (SEM)

BV-2 cells were cultured on 10 mm glass coverslips coated with 1% poly-l-lysine and fixed for 1–2 h in 2% paraformaldehyde and 3% glutaraldehyde in PBS buffer. The fixed BV-2 microglial cells were washed in PBS buffer, post-fixed for 30 min in 1% OsO_4_, and dehydrated through ethanol series, then were critical-point dried, mounted on stubs, sputter coated with a thin layer of conductive metal, gold and palladium, and viewed under SEM (FEI Quanta 650 FEG SEM system).

### Transmission Electron Microscopy (TEM)

BV-2 cells were fixed in 2% paraformaldehyde and 3% glutaraldehyde in 0.1 M PBS overnight at 4 °C and post-fixed with 1% OsO_4_ for 1.5 h and rinsed twice with water for 5–10 min at room temperature (RT). The samples were then dehydrated through an ascending grade series of ethanol at RT (25% for 10 min, 50%, 75%, 95%, 100% at 20 min each) and then 100% acetone for 20 min twice. The samples then infiltrated with increasing the concentration of hydrophilic resin and araldite 502 as follows: 30 min in 100% acetone:resin (1:1) at RT, 24 h 100% acetone:resin (1:6) at RT, 20 min in fresh resin at RT, 30 min in fresh resin at 40 °C, and 1 h in fresh resin at 45 °C twice. The resin-infiltrated specimens were then transferred into molds containing fresh resin and polymerized at 60 °C for at least 24 h. Semi-thin (1 μm thickness) sections were obtained before continuing to ultra-thin sections (Gold interference color) collected onto the copper grids. Each grid was stained with uranyl acetate and examined using Joel 1010 transmission electron microscope and Gatan micrograph software.

### Western Blot Analysis

BV-2 cells were treated with vehicle, 200 μM DHA, or 200 μM cisplatin for 2 h. Whole-cell lysates were prepared in RIPA lysis buffer. Lysates were then spun at 10,000 rpm for 10 min to remove insoluble material and protein content in the lysates was measured by Bio-Rad protein assay dye reagent concentrate (Bio-Rad, USA). Lysates were electrophoresed on a 10% SDS gel, electrotransferred to a nitrocellulose membrane, blocked with Blocking One (Nacalai Tesque, Inc., JAPAN) blocking buffer, and probed with a rabbit polyclonal antibody to Gsdmd (Cell Signaling Technologies #93709) overnight at 4 °C. The blot was then washed with TBS with 0.1% Tween-20 and exposed to anti-rabbit–horseradish peroxidase (HRP) conjugate for 1 h, then examined by Western Bright Sirius HRP substrate (Advansta). Three independent experiments were performed and run on the same gel for analysis. Images were captured using Chemidoc XRS + imaging system and analyzed using Image Lab software (Bio-Rad, USA). Band density was quantified with ImageJ.

### Flow Cytometry

Cells were collected by gentle trypsinization, washed with FACS buffer, fixed with the eBioscience Fixation/Permeabilization buffer for 15 min at RT and washed once in 1× Permeabilization buffer. For intracellular staining, cells were stained with anti-iNOS-APC/Cy7 antibody (eBioScience) and anti-Arg1-FITC (R&D) for 30 min at RT. After washing once in 1× Permeabilization buffer, cells were resuspended in FACS buffer and run on a BD LSRFortessa Flow Cytometer (BD, NJ). Data were analyzed with FlowJo (Treestar, OR).

### Statistical Analysis

All tests for significance were performed by two-way ANOVA with Tukey’s multiple comparison test. Differences were considered significant when *P* < 0.05.

## Results

**Fig. 1:**
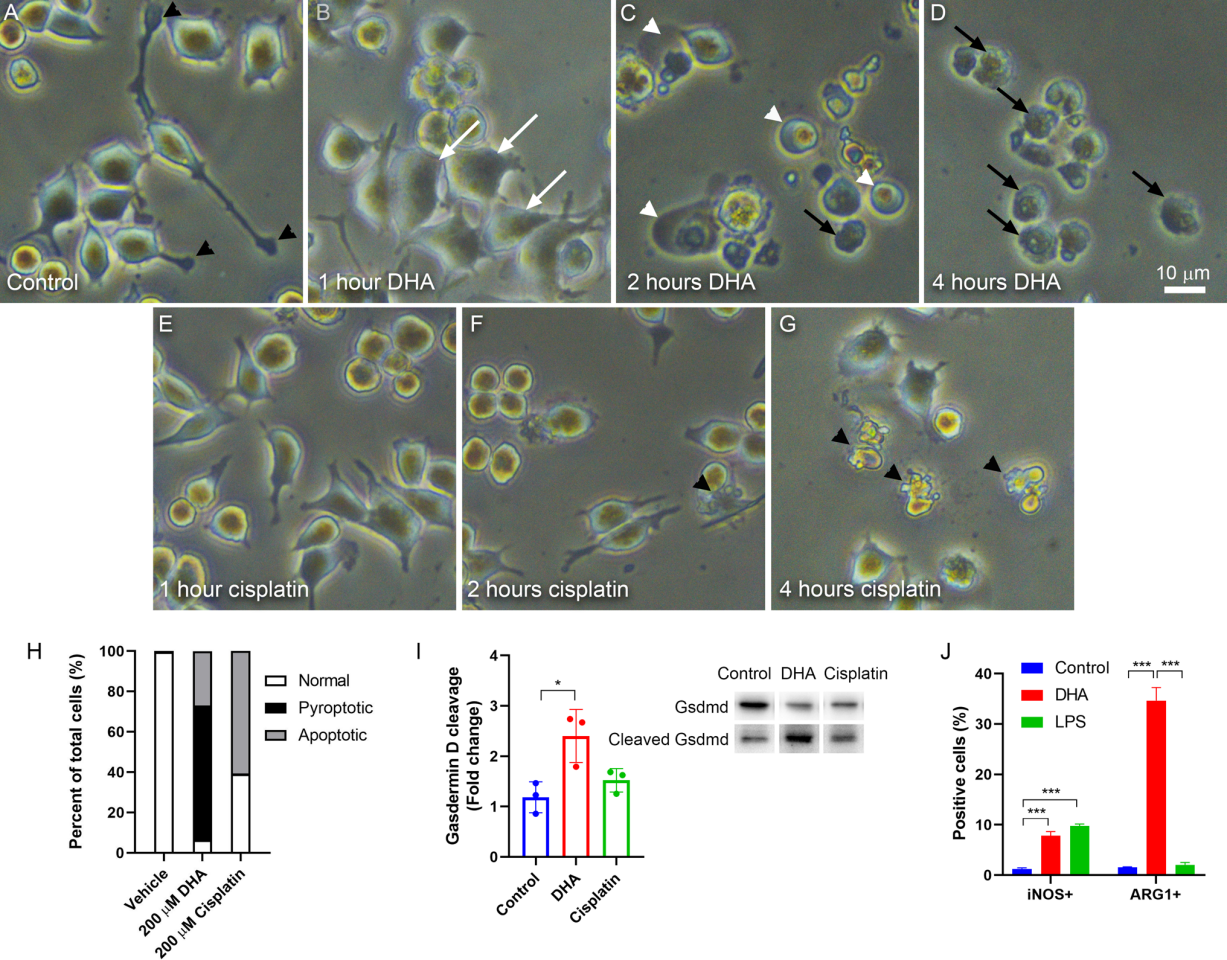
DHA activates morphological and molecular characteristics of pyroptosis. **a**–**d** Phase-contrast photomicrographs showing BV-2 microglial cells treated with 200 μM DHA. **a** Untreated cells appear normal with extended processes and active filopodial tips (black arrowheads). **b** By 1 h after DHA treatment, cells are larger with shorter processes and apparent swelling of the cell bodies (white arrows). **c** At 2 h after DHA treatment, cell processes are absent. Many cells have a balloon-like appearance (white arrowheads), while others appear collapsed with irregular boundaries (black arrows). **d** By 4 h after DHA treatment, nearly all cells appear collapsed. **e**–**g** Phase-contrast photomicrographs showing BV-2 microglial cells treated with 200 μM cisplatin. **e** At 1 h after cisplatin treatment, cells are largely unchanged. **f** After 2 h of cisplatin treatment, there is some loss of cellular processes and increased cell rounding. Some cells display membrane blebs that are characteristic of apoptosis (black arrowheads). **g** By 4 h after cisplatin treatment, most cells appear apoptotic. Scale = 10 μm (**a**–**g**). **h** Quantification of morphologies observed following 2 h of treatment with 200 μM DHA or 4 h with 200 μM cisplatin. **i** Quantification and representative western blot of full-length and cleaved Gasdermin D (Gsdmd) for BV-2 cells treated with vehicle, 200 μM DHA, or 200 μM cisplatin for 2 h. Quantification depicts the ratio of cleaved Gsdmd to full-length Gsdmd relative to vehicle-treated control cells. **j** Flow cytometry analysis of BV-2 cells treated with vehicle, 200 μM DHA, or 1 μg/ml LPS for 2 h. Quantification depicts % cells positively labeled for iNOS (M1) or ARG1 (M2). *N* = 3, *P < 0.05, ***P < 0.001

Untreated BV-2 microglial cells demonstrated normal appearance by phase-contrast microscopy, including characteristics such as extended processes with active filopodial tips (Fig. 1a). Within 1 h of exposure to 200 μM DHA, cells exhibited a loss of filopodial tips, retraction of processes, and cell swelling manifesting as an apparent increase in cell size (Fig. 1b). Within 2 h of exposure, cell swelling has become pronounced, leading many cells to rupture and collapse (Fig. 1c). By 4 h, nearly all cells have collapsed, with irregular plasma membranes and exposed cytoplasmic debris (Fig. 1d). This response contrasts that obtained from exposure to 200 μM cisplatin (Fig. 1e–g). The cisplatin-mediated response occurs at a slower rate, showing no apparent morphological change at 1 h (Fig. 1e), some evidence of process retraction and apoptotic morphology at 2 h (Fig. 1f), and widespread apoptosis only at 4 h (Fig. 1g). The morphology of cisplatin-induced apoptosis is markedly different from that of DHA-induced pyroptosis, in that cisplatin results in the formation of large membrane blebs and fragmentation of the cell body (Fig. 1f–g). Quantification of these morphologies reveals that 200 μM DHA treatment predominantly results in pyroptotic morphologies, but ~ 25% of cells appear apoptotic. There is no evidence of pyroptosis in cisplatin-treated cells (Fig. 1h).

To further confirm our previous observation that the DHA-mediated response was due to the activation of pyroptosis (Srikanth et al. 2018), we evaluated DHA-treated cells for cleavage of Gsdmd, a hallmark of pyroptosis (Aglietti et al. 2016). Indeed, treatment of BV-2 cells with 200 μM DHA for 2 h resulted in a significant increase of Gsdmd cleavage, a response that was absent when cells were treated with 200 μM cisplatin (Fig. 1i, Supp. Fig. S2). To determine whether this response affected the polarization of these macrophage-like cells, we evaluated the expression of iNOS (M1 marker) and ARG1 (M2 marker) following DHA treatment. Interestingly, we observed a significant increase in iNOS-positive cells and a marked increase in ARG1-positive cells (Fig. 1j, Supp. Fig. S1). Notably, lipopolysaccharide (LPS) is also able to induce an M1-like phenotype, confirming that these BV-2 cells represent physiological-relevant models of microglial cells.

**Fig. 2:**
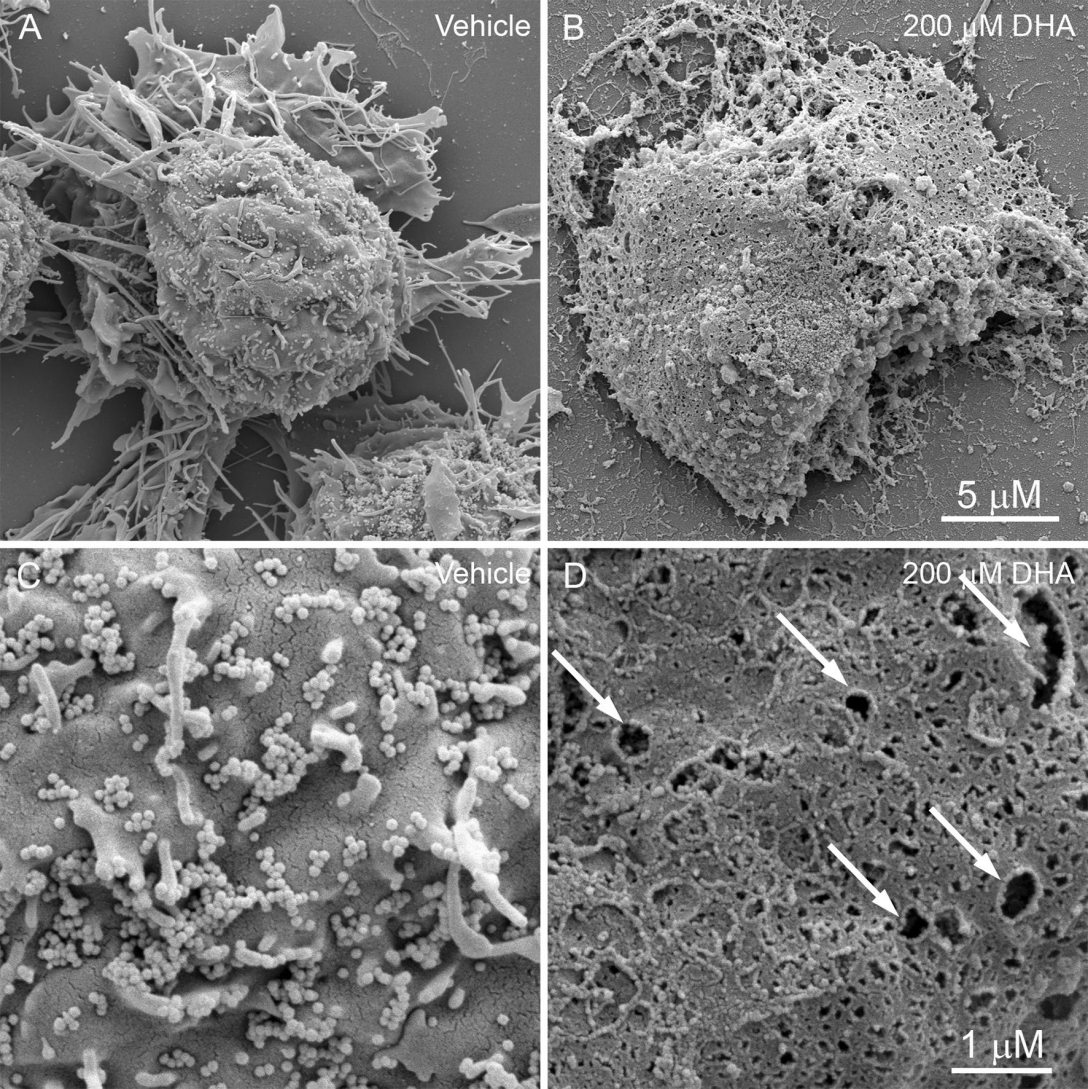
Scanning electron micrographs of BV-2 cell 4 h after treatment with vehicle (**a**, **c**) or 200 μM DHA (**b**, **d**). **a** At × 12,000 magnification, control cells are characterized by a rounded cellular mass with extended cellular processes. **b** DHA-treated cells have irregular margins and lack processes. The plasma membranes appear ruptured with extrusion of cellular contents. **c** At × 50,000 magnification, the surface of control cells exhibit projections from the plasma membrane and tethered extracellular vesicles (EVs). **d** DHA treatment causes the formation of membrane pits and pores of varying sizes, and a pronounced loss of EVs. Scale = 5 μm (**a**, **b**) and 1 μm (**c**, **d**)

BV-2 cells were next evaluated for detailed surface morphology by SEM after vehicle or 200 μM DHA treatment (Fig. 2). Vehicle-treated cells were characterized by extended processes, spine-like projections (Fig. 2a), and numerous extracellular vesicles (EVs) tethered to the surface of the plasma membrane (Fig. 2c). Long, slender cytoplasmic extensions were observed on the cell surface, which vary from approximately 0.4 to 5.2 µm in length (Fig. 2a, c). The EVs resemble small, spherical extensions of the cell membrane of fairly uniform diameter of approximately 90 nm (Fig. 2c). In contrast to vehicle-treated cells, gross abnormalities were observed after treating cells with 200 µM DHA for 4 h (Fig. 2b, d). The most prominent of these abnormalities is the appearance of numerous pits or pores of varying sizes across the cell surface (Fig. 2d), which is associated with structural collapse and flattening of the cell shape (Fig. 2b). The cellular processes were completely retracted giving the cells a rounded morphology (Fig. 2b). Moreover, the EVs and spines were completely absent following 200 μM DHA treatment, possibly due to release from the cell surface and contributing to the accumulation of extracellular debris (Fig. 2b, d).

**Fig. 3:**
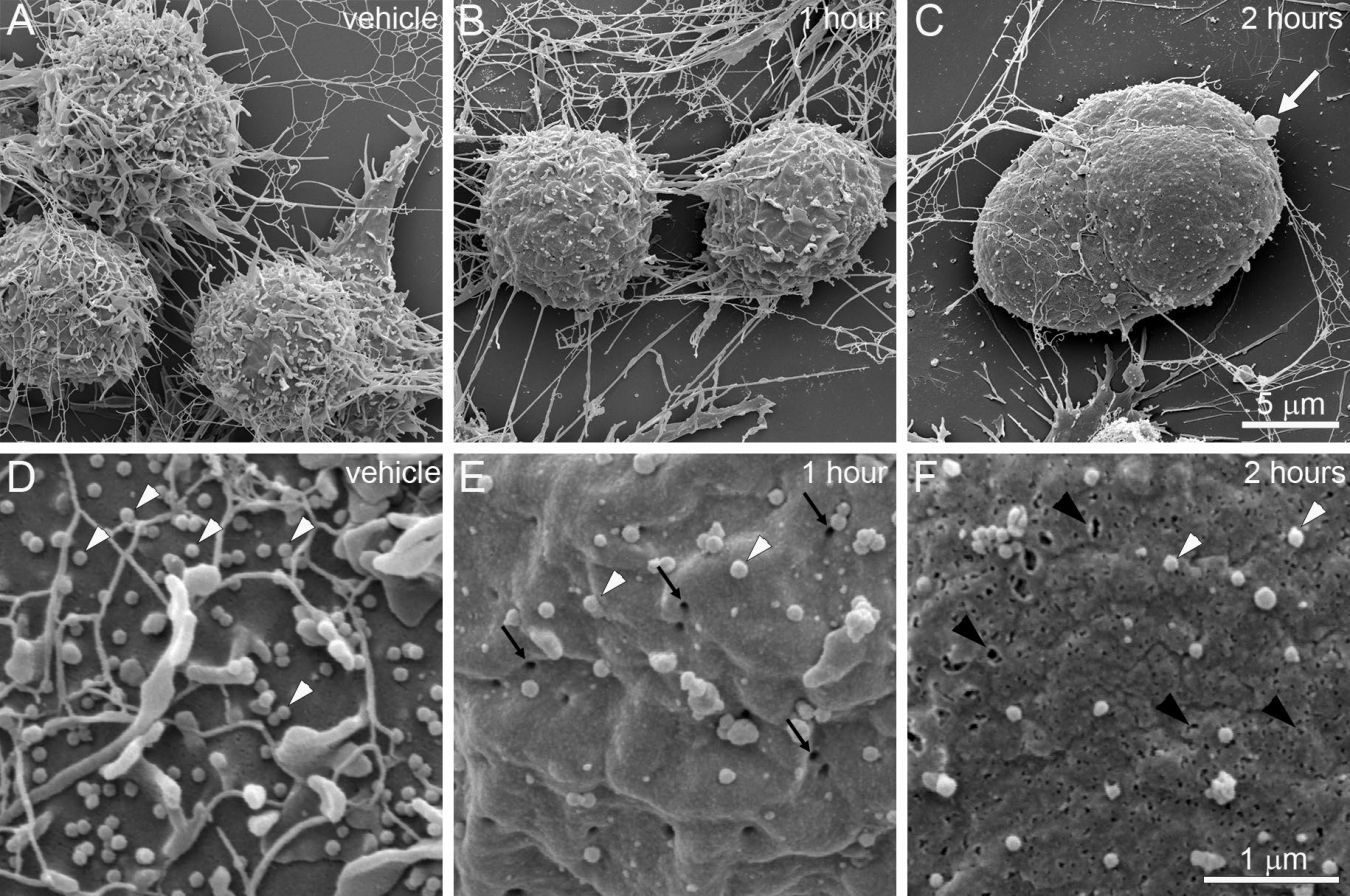
Time course of DHA-induced ultrastructural changes observed by SEM. **a**–**c** Images were captured at × 12,000. **d**–**f** Images were captured at × 50,000. ** a** Vehicle-treated BV-2 cells exhibit normal morphology. **b** After 1 h, cells show loss of processes and cytoplasmic extensions. **c** At 2 h, cells exhibit irregular bulges and occasional membrane blebs (white arrow). **d** Higher magnification of vehicle-treated cells reveals numerous EVs on the cell surface (white arrowheads). **e** There is a significant loss of EVs at 1 h after DHA treatment, accompanied by the appearance of membrane pores (black arrows). **f** By 2 h, membrane pores become enlarged and irregular (black arrowheads). Scale bars apply to all panels in each row

To determine the sequence and timeline of these ultrastructural changes, BV-2 cells were observed by SEM 1 h and 2 h after DHA treatment (Fig. 3). Consistent with the characteristics revealed by light microscopy, cells appeared more rounded with loss of extended processes (Fig. 3b). In addition, higher magnification revealed extensive loss of EVs and cytoplasmic extensions at this stage, and the appearance of numerous, homogenous membrane pores of approximately 30 nm in diameter (Fig. 3e). By 2 h, many cells exhibited bulging masses and occasional blebs that resemble reported “pyroptotic bodies” (Chen et al. 2016) (Fig. 3c). Cytoplasmic extensions became nearly absent, along with further loss of EVs. In addition, membrane pores became larger, irregular, and heterogeneous in size (Fig. 3f).

**Fig. 4:**
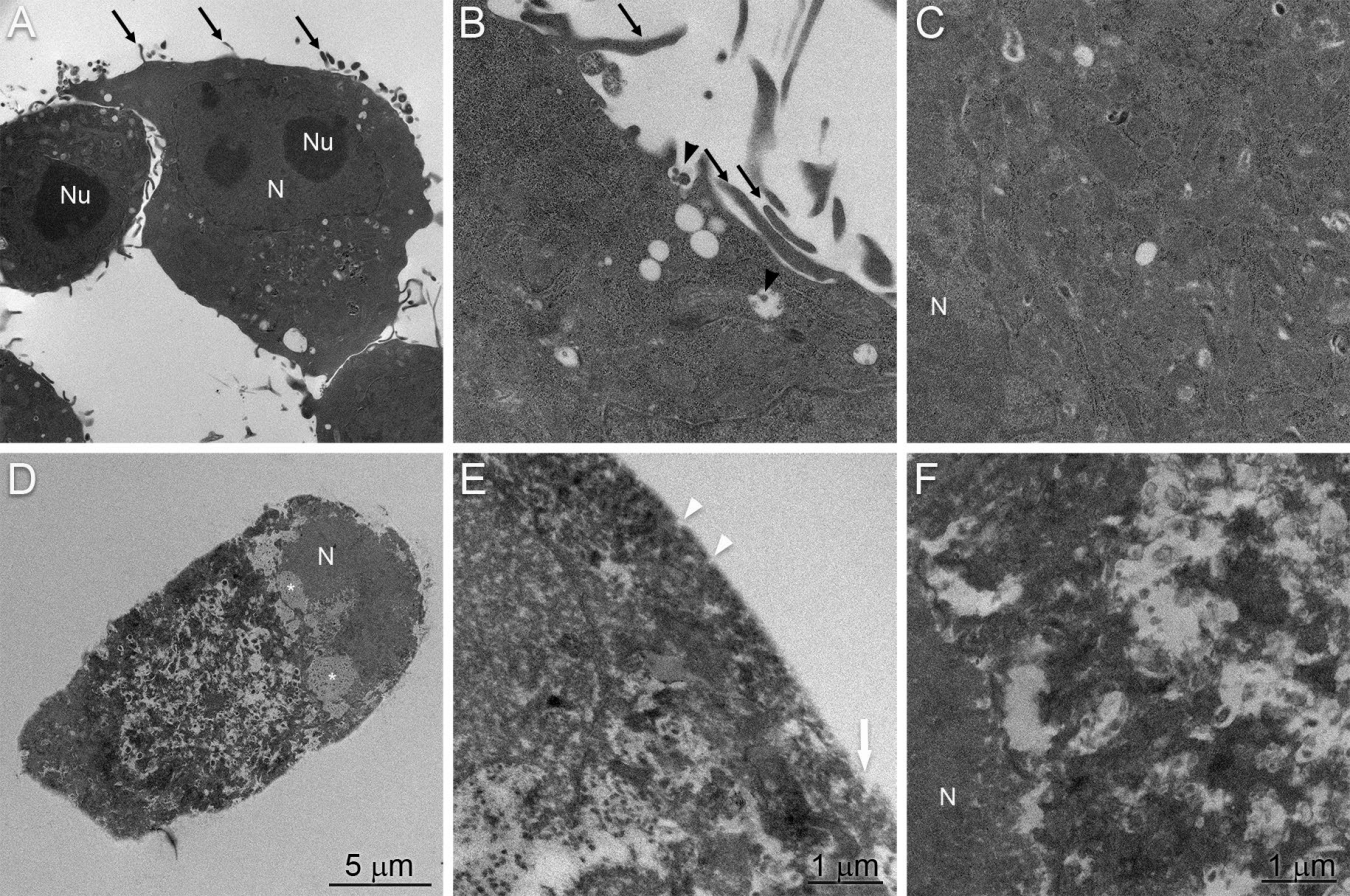
Transmission electron micrographs of BV-2 cell 4 h after treatment with vehicle (**a**–**c**) or 200 μM DHA (**d**–**f**). **a** Control cells have irregular outlines. The nuclei are intact and the cytoplasm contains a number of cytoplasmic organelles with a few small vacuoles. **b** The cell surface shows an intact plasma membrane with cytoplasmic protrusions (black arrows) and tethered extracellular vesicles (black arrowheads). **c** The cytoplasm is ordered with intact organelles and an intact nuclear envelope. **d** 4 h after treatment with 200 uM DHA, BV-2 cells become rounded and the nuclei become diffuse with regions of low electron density (asterisks). **e** The cell surface lacks complex features and contains membrane pores (white arrowheads) and disrupted regions (white arrow). **f** The nuclear envelope is characterized by large disruptions and the cytoplasm lacks discernable organelles. Scale bars apply to both panels in each column. *N* nucleus, *Nu* nucleolus

We also evaluated BV-2 cells by TEM to identify any intracellular structures that are characteristic of pyroptosis. Vehicle-treated BV-2 cells showed the expected normal characteristics of microglial cells, such as dark nuclei with marginated heterochromatin, dark cytoplasm with cytoplasmic organelles (Fig. 4a–c). The mean size of the nuclei was approximately 6.9 µm and the nucleoli were approximately 3.5 µm. As with the SEM analysis, cells were characterized by cytoplasmic extensions and tethered extracellular vesicles that were also apparent inside intracellular multivesicular bodies (Fig. 4b). The cytoplasm contained well-defined organelles and an intact nuclear envelope. Treatment of BV-2 microglial cells with 200 μM DHA resulted in complete loss of identifiable organelles (Fig. 4d). The plasma membrane was characterized by numerous pores and large disruptions (Fig. 1e). The nucleus remained identifiable but lost envelope integrity and became largely diffuse with regions low electron density (Fig. 4d, f).

**Fig. 5:**
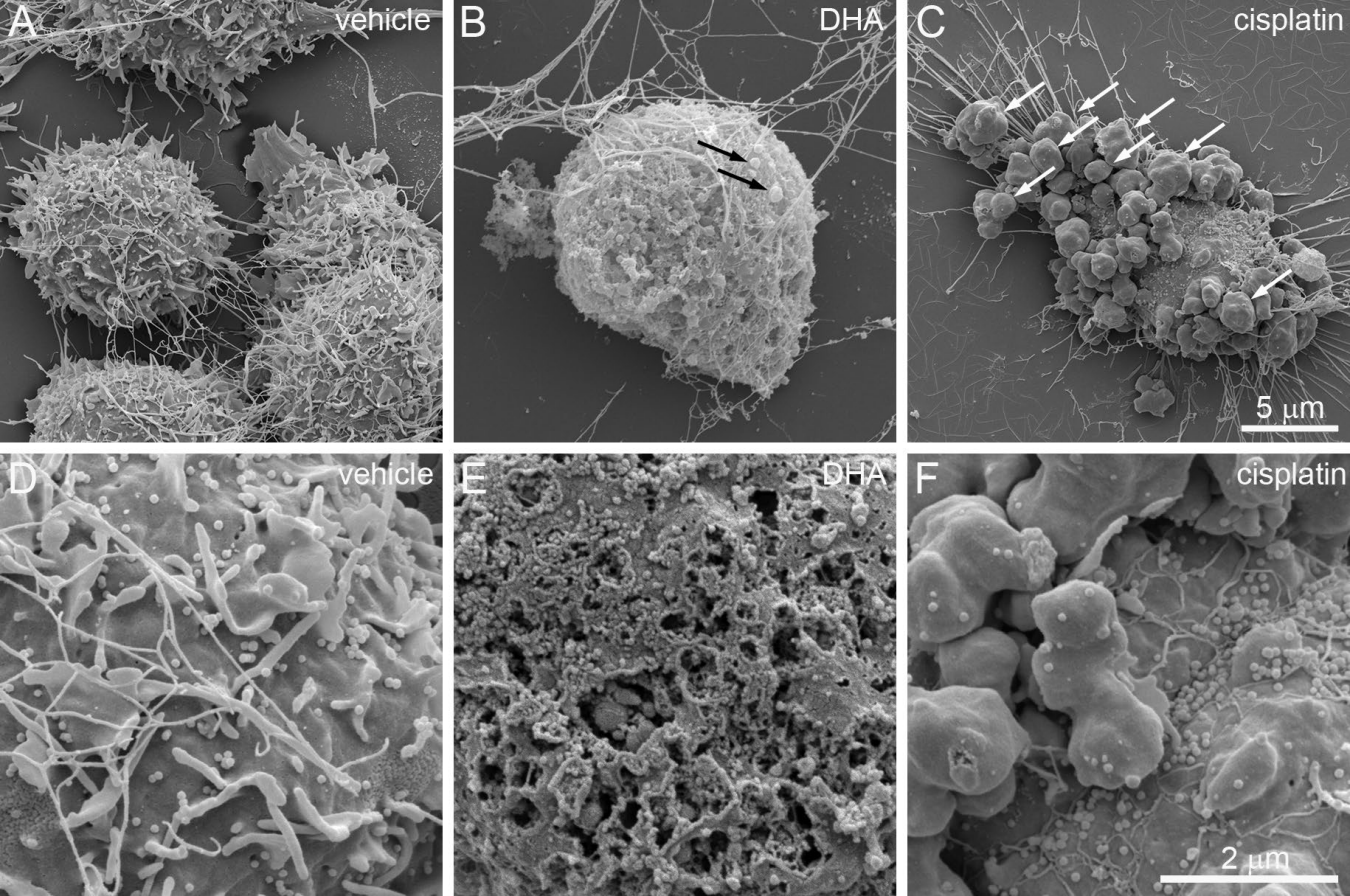
Comparison of DHA-induced pyroptosis and cisplatin-induced apoptosis by TEM. BV-2 cells were treated for 4 h with vehicle (**a**, **d**), 200 μM DHA (**b**, **e**), or 200 μM cisplatin (**c**, **f**). **a** Vehicle-treated cells show normal morphology with extended processes. **b** DHA-treated cells exhibit swelling, rounding, and appearance of occasional pyroptotic bodies (black arrows). **c** Cisplatin-treated cells are characterized by prominent membrane blebs (white arrows). **d** At higher magnification (× 50,000), vehicle-treated cells have intact plasma membranes with abundant EVs. **e** DHA-treated cells have a pronounced loss of EVs and the appearance of membrane pores. **f** The membranes of cisplatin-treated cells largely maintain EVs despite the emergence of apoptotic bodies. Scale bars apply all panels in each row

**Fig. 6:**
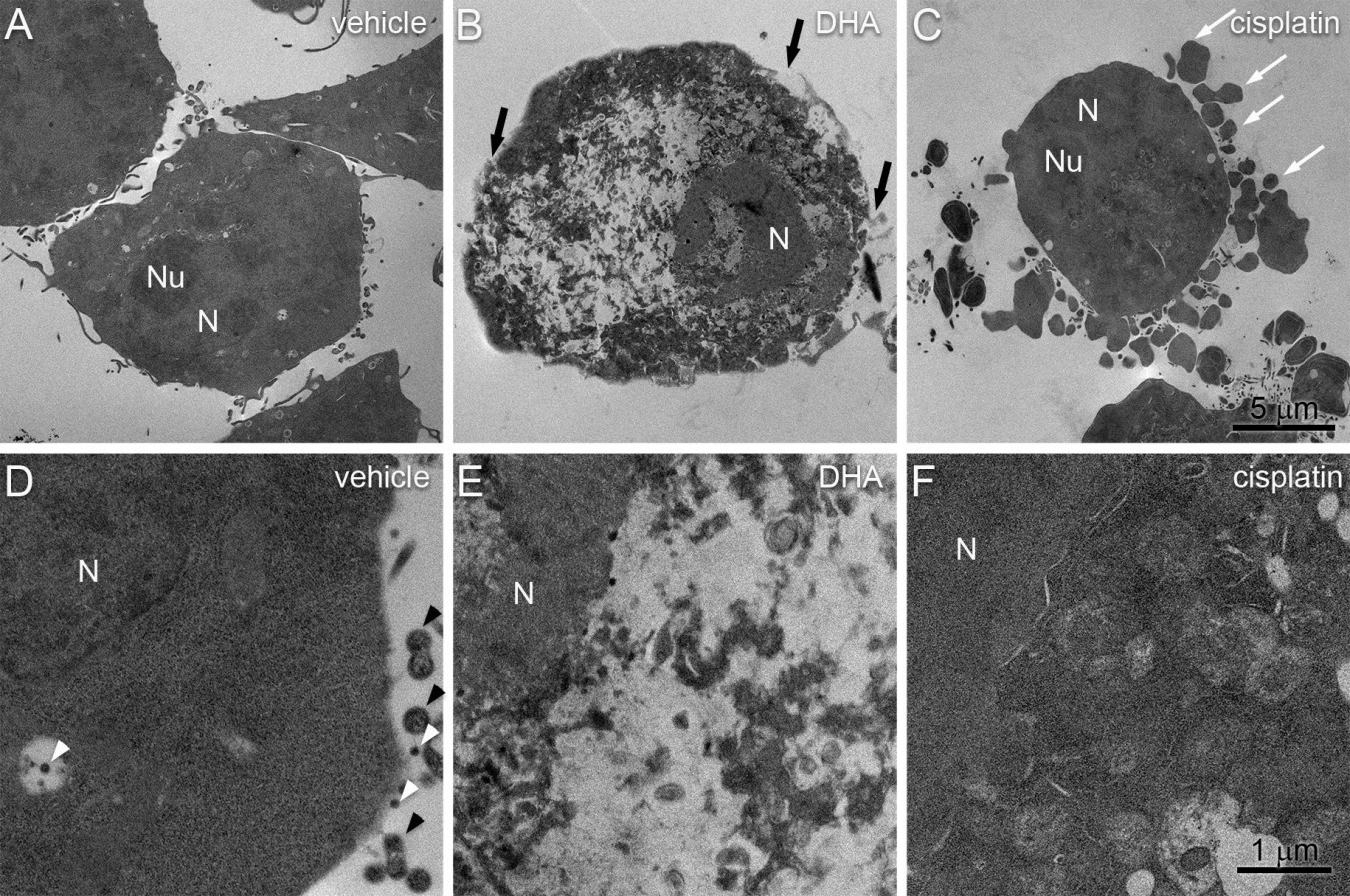
Comparison of DHA-induced pyroptosis and cisplatin-induced apoptosis by SEM. BV-2 cells were treated for 4 h with vehicle (**a**, **d**), 200 μM DHA (**b**, **e**), or 200 μM cisplatin (**c**, **f**). **a** Vehicle-treated cells show intact cellular morphologies with structured nuclei and cytoplasmic protrusions. **b** DHA-treated cells exhibit loss of cytoplasmic and nuclear organization, loss of cell processes, and disruptions of the plasma membrane (black arrows). **c** Cisplatin-treated cells were again characterized by prominent membrane blebs (white arrows) while maintaining apparent cytoplasmic and nuclear organization. **d** At higher magnification, vehicle-treated cells have intact plasma membranes with cytoplasmic protrusions (black arrowheads) and EVs (white arrowheads). **e** DHA-treated cells are again characterized by nuclear envelope disruptions and a lack of discernable organelles. **f** The nuclear envelope and cytoplasmic organelles of cisplatin-treated cells remain largely intact. Note that organelles are more tightly packed and have decreased electron density compared to vehicle-treated cells. Scale bars apply all panels in each row

To contrast the ultrastructural changes of DHA-induced pyroptosis with those of apoptosis, DHA-treated cells were compared to cells treated with a known inducer of apoptosis, cisplatin (Fig. 5) (Barry et al. 1990; Herr et al. 2016). Notably, cisplatin treatment resulted in the appearance of pronounced membrane blebs that resemble canonical apoptotic bodies (Fig. 5c). Interestingly, this was not associated with the extensive loss of EVs (Fig. 5f) that is observed with DHA treatment (Fig. 5e). TEM analysis revealed pronounced intracellular differences between these processes (Fig. 6). Compared to vehicle-treated control cells (Fig. 6a, d), DHA-treated cells were characterized by loss of plasma membrane integrity, disorganization throughout the cytoplasm, and disruption of the nuclear envelope (Fig. 6b, e). By contrast, cisplatin-treated cells retained intact nuclear envelopes and membrane-bound organelles, but the nuclear envelope was discontinuous and the organelles were tightly packed and were characterized by loss of electron density (Fig. 6c, f).

## Discussion

This study was performed to provide the first detailed ultrastructural analysis of BV-2 microglial cells undergoing cisplatin-induced apoptosis and DHA-mediated pyroptosis. Microglia are a type of glial cells located throughout the brain and spinal cord. As the resident macrophage cells, they act as the first and main form of active immune defense and mediate inflammatory functions such as the release of cytokines. Under phase-contrast microscopy, vehicle-treated microglia were observed to have clear cytoplasm and extended processes. By SEM, the cells were observed to have finger-like processes and very small blebs resembling EVs observed on the surface. These blebs are likely to be exosomal vesicles (Raposo and Stoorvogel 2013) that are known to be present in immune cells. They have been demonstrated to selectively incorporate cargo such as mRNA, miRNA, and a large variety of other small noncoding RNA species (Nolte-’t Hoen et al. 2012). Interestingly, it has been shown that the cargo in these vesicles can be functionally transferred as a consequence of fusion with recipient cells (Mittelbrunn et al. 2011; Montecalvo et al. 2012). Therefore, it is likely that there is functional significance to the DHA-mediated release of EVs from microglia.

These ~ 90 nm EVs are very different in size from the large membrane blebs (> 1 μM) that we observed on these cells after treatment with cisplatin, which is known to exert its clinical effects by inducing apoptosis (Barry et al. 1990). The cisplatin-induced blebs resemble the structural characteristics of apoptotic bodies, a feature found on cells undergoing apoptosis. Apoptotic bodies not only accumulate a collection of important “eat-me” factors such as phosphatidylserine and C1q, but also appear to be rich in auto-antigens such as DNA, nucleosomes, and nuclear antigens, e.g., Ro and La (Fadeel 2004; Paidassi et al. 2011; Schiller et al. 2008). One of the key characteristics of apoptotic cells that allows for the rapid and stealthy removal of cellular fragments is a stable intact membrane that prevents release of intracellular proteins and consequent immunological activation (Overbeeke et al. 1998). However, if apoptotic bodies are not efficiently engulfed by macrophages, they persist as undigested apoptotic debris and have the potential of inducing autoimmune responses (Wickman et al. 2012). More research needs to be carried out on a possible role of “microglial apoptotic bodies” in autoimmune CNS diseases such as amyotrophic lateral sclerosis (ALS) or experimental allergic encephalomyelitis (EAE).

After treatment with 200 µM DHA, BV-2 cells were observed under phase-contrast microscopy to exhibit decreased membrane ruffling, activation of process retraction, and pronounced cell rounding. Cells then exhibited a gradual increase in swelling of both cytoplasm and nucleus. These features are similar to canonical pyroptotic responses that occur in cultured macrophages infected with Salmonella spp. (Chen et al. 1996). The final state of this process was characterized by a decrease in size due to cell lysis, accompanied by the release of cytoplasm into the extracellular space. By SEM, microglial cells that were exposed to 200 µM DHA showed absence of finger-like processes and EVs, and instead had a large number of pores on the cell membrane. The pores were initially a uniform ~ 30 nm in diameter, consistent with the previously reported 15–32 nm GSDMD pore complexes (Liu et al. 2016), but grew larger and increased in number over time. The appearance of these pores is consistent with those that occur on immune antigen-presenting cells of the monocyte/macrophage lineage following the activation of PRRs by microbial-derived ligands (Liu and Lieberman 2017). These pores then allow for the release of inflammatory cytokines such as interleukin-1β to the extracellular space (Shi et al. 2015; Kayagaki et al. 2015). Our previous work demonstrated that treatment of BV-2 cells with 200 μM DHA induces molecular phenomena that are similar to these microbe-driven inflammatory responses (Srikanth et al. 2018).

The increased expression of ARG1 (Fig. 1f) suggests that DHA may promote an M2-like phenotype in these cells, and that some of the observed phenotypes (i.e., process retraction and rounding) may reflect cell polarization rather than a proximal effect of the activation of pyroptosis. Further study is needed to understand the physiological significance of this phenomenon.

It is important to note that the majority of DHA in the brain is sequestered in phospholipids as a structural component of the plasma membrane (Lauritzen et al. 2001) and that the concentration of *free* DHA is quite low. However, it is likely that the effect produced here remains physiologically relevant. DHA is the most abundant fatty acid in the brain, comprising ~ 18% of the fatty acid content of membrane phospholipids (Calder 2016). This serves as a large reservoir that can be rapidly released by the activity of calcium-independent phospholipase A2 (iPLA_2_) (Yang et al. 2019). It is likely that induction of iPLA_2_ could result in local concentrations of DHA that exceed the 50 µM needed for activation of pyroptosis (Srikanth et al. 2018). Furthermore, since lipoxygenase activity is required for DHA-induce pyroptosis (Srikanth et al. 2018), we conclude that a DHA metabolite, rather than DHA itself, is the proximal bioactive ligand. Signaling concentrations of this metabolite are presumably significantly lower than 50 µM, and its production may not require high free DHA concentrations in vivo.

Cumulatively, this manuscript describes the ultrastructural changes that occur in BV-2 microglial cells following treatment with 200 µM DHA and provides morphological data to complement our biochemical results (Srikanth et al. 2018) that show that pyroptosis can be induced by DHA in a microglial cell line. The functional implications of this process are still unknown. It is possible that induction of pyroptosis by DHA metabolites represents a novel initiating pattern of sterile inflammation in the CNS (Rubartelli et al. 2013), for example, in ischemic stroke (Poh et al. 2019). Alternatively, pyroptotic death of microglia may serve to limit chronic inflammation through loss of pro-inflammatory immune cells (Ortega-Gomez et al. 2013). More research is needed to determine whether high dose DHA-induced microglial pyroptosis has a net pro- or anti-inflammatory effect in the brain, and consequences in brain infection, cancer, and neuroinflammatory diseases.

## Electronic supplementary material

Below is the link to the electronic supplementary material.

Supplementary material 1 (PDF 408 kb)

## Abbreviations

CNS: Central nervous system
DHA: Docosahexaenoic acid
EV: Extracellular vesicle
PCD: Programmed cell death
PRR: Pattern recognition receptors
SEM: Scanning electron microscopy
TEM: Transmission electron microscopy
