# Supplementary material for: Ultrastructural Characteristics of DHA-Induced Pyroptosis
Source: Neuromolecular Med. 2020 Jan 4;22(2):293–303. doi: 10.1007/s12017-019-08586-y (PMC7230060; doi:10.1007/s12017-019-08586-y)
Supplement: Supplementary file 1 — Supplementary material 1 (PDF 408 kb) [file 12017_2019_8586_MOESM1_ESM.pdf]

Supplementary Material

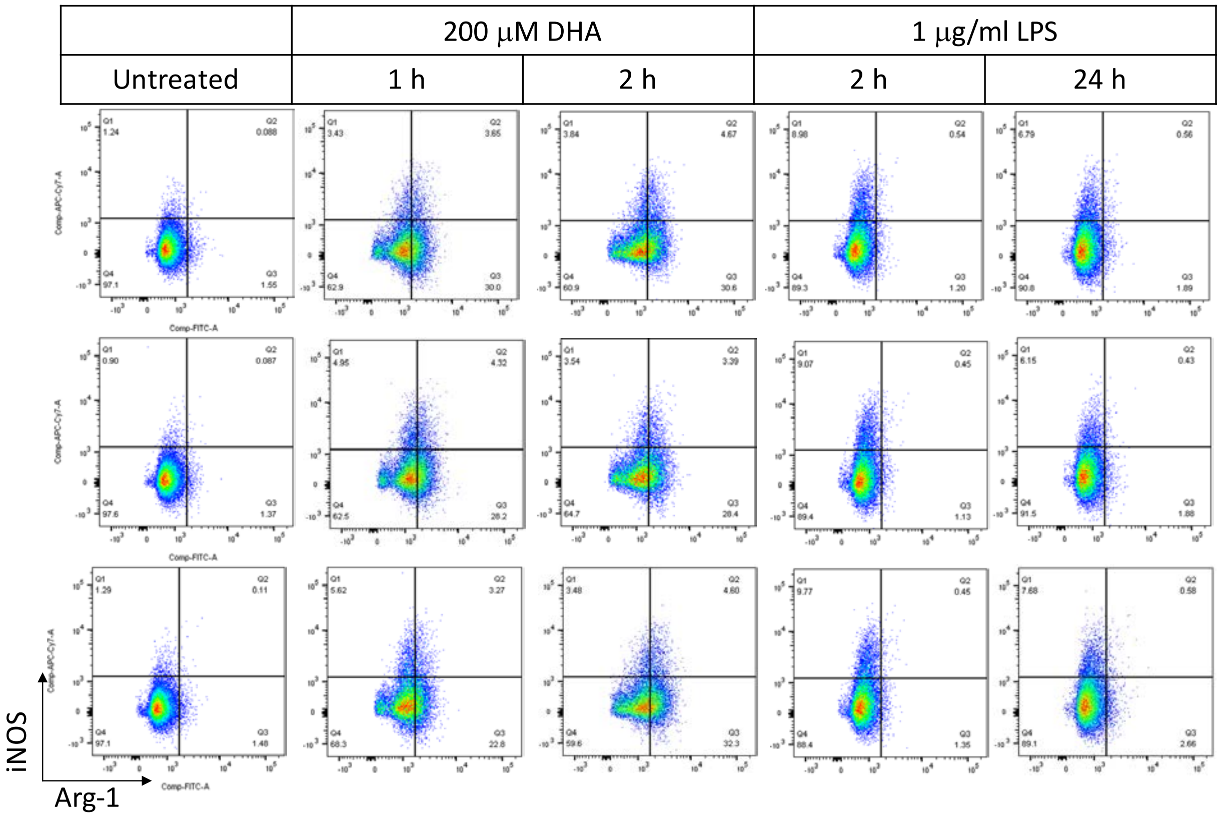

Supplemental Figure S1: Cells were treated with DHA or lipopolysaccharide (LPS) as indicated, then evaluated by flow cytometry for iNOS and ARG-1.

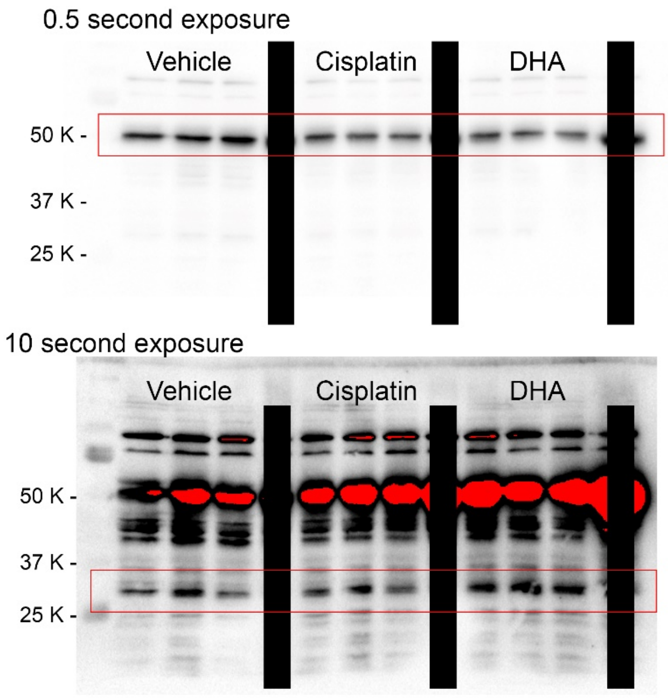

Supplemental Figure S2: Unmodified western blots. Cells were treated with vehicle, 200 µM DHA, or 200 µM cisplatin for 2 hours before collecting and evaluating for gasdermin D. Full length gasdermin D is apparent after 0.5 second exposure (upper panel, red box) while the cleaved fragment required a 10 second exposure for visualization (lower panel, red box).
